# A Call to Include a Perspective of Sustainable Development in Physical Therapy Research

**DOI:** 10.1093/ptj/pzaa228

**Published:** 2020-12-31

**Authors:** Annie Palstam, Mathias Andersson, Elvira Lange, Anton Grenholm

**Affiliations:** Institute of Neuroscience and Physiology, Sahlgrenska Academy, University of Gothenburg, Gothenburg, Sweden; Department of Rehabilitation Medicine, Sahlgrenska University Hospital, Gothenburg, Sweden; School of Education, Health and Social Studies, Medical Science, Dalarna University, Falun, Sweden; School of Education, Health and Social Studies, Medical Science, Dalarna University, Falun, Sweden; Smärtrehabilitering Säter/Smärtmottagning Falun, Region Dalarna, Sweden; Department of Health and Rehabilitation, Unit of Physiotherapy, Institute of Neuroscience and Physiology, The Sahlgrenska Academy, University of Gothenburg, Gothenburg, Sweden; Region Västra Götaland, Research and Development Primary Health Care, Gothenburg, Sweden; Närhälsan Eriksberg Rehabilitation Centre, Region Västra Götaland, Sweden; Office of Education and Research, Dalarna University, Falun, Sweden

**Keywords:** Physical Therapists, Professional Issues, Quality of Health Care, Environmental Health

The health sector has an important role to play in the work for sustainable development, where the opportunity for physical therapy to take an essential part must be considered and further evaluated. In this call, we adhere to the widely used definition of sustainable development first described in the 1987 UN report, Our Common Future: “Sustainable development is development that meets the needs of the present without compromising the ability of future generations to meet their own needs.”^1^

**Figure 1:**
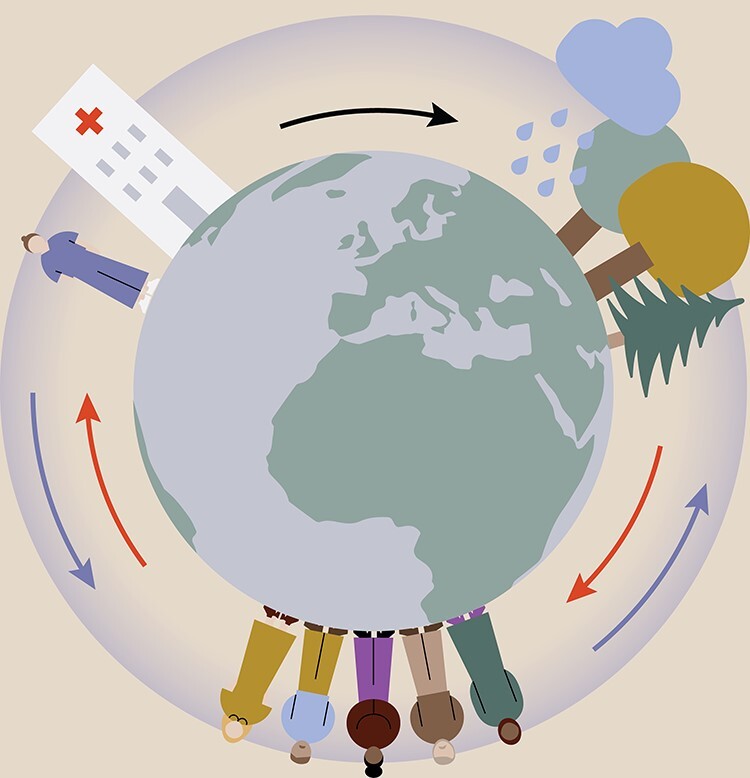
Model for causal relationships between the health sector, the climate and environmental systems, and human health and lifestyles (A. Grenholm, illustration J. Wallhult). Global environmental changes, such as climate change and loss of biodiversity, greatly impact human health and well-being, hence increasing and altering the demands on the health sector (red arrows). Furthermore, the health sector contributes to significant negative effects on the environment through large consumption of energy and resources as well as pollution caused by pharmaceuticals, among other things (black arrow). At the same time, the health sector has great opportunities to contribute to reduced human impact on the environment. Here, physical therapy plays an important role, through providing treatment options that reduce the need for resources and pharmaceuticals and by promoting behavioral changes that have positive effects on health as well as the environment (blue arrows). The occurrence and magnitude of these effects are relevant to include and evaluate in physical therapy research.

Physical therapy could be a vital contributor to sustainable development at different levels and for many reasons. For example, support for behavioral change and patient empowerment is central to physical therapy, and lifestyle changes that favor one’s own health are often favorable from a climate and resource perspective as well. Further, physical therapy is a non-pharmacological and resource-efficient option that potentiates other forms of treatment, thereby offering the possibility to significantly reduce the environmental burden of health care. We therefore argue that it is essential to include a perspective of sustainable development in physical therapy research to better understand and take full advantage of the environmental and societal co-benefits that physical therapy holds.

In this Point of View, we will describe different causal relationships between the health sector, the climate and environmental systems, and human health and lifestyles that effect, and are affected by, these systems (Fig. 1).

The Lancet Commission on Health and Climate Change states that climate change constitutes the greatest global threat to human health during this century,^2^ and already the consequences of climate change and air pollution affect the health of people worldwide.^3^ Beyond climate change, there are a number of areas in which increasing human impact is pushing the limits for what essential biophysical processes can manage while still being able to continue supporting human prosperity. Here, 9 planetary boundaries have been identified, that is, theoretical limits on changes to the environment that should not be transgressed if we are to avoid unacceptable global environmental change. It was estimated that 4 of the 9 boundaries—climate change, loss of biodiversity, land utilized by humans, and altered biogeochemical cycles—have already been crossed.^4^ These global environmental changes will have great impact on human health and well-being in the coming decades and will thereby cause profound challenges for the health sector (Fig. 1, red arrows).

To take on the global challenges of today, the 2030 Agenda for Sustainable Development was adopted by all the member states of the United Nations in 2015.^5^ The agenda includes 17 Sustainable Development Goals (SDGs), defined in 169 targets. These goals are integrated and inseparable and include all 3 domains of sustainability: economic, social, and environmental. To achieve these goals and targets, commitment is required not only from governments; efforts must be in collaboration with the public as well as private sectors, academia, and the civil society.^6^ We believe that physical therapy already, although not pronounced, plays an important role in this work. Relationships between physical therapy and the SDGs need to be highlighted, further developed, and evaluated to uncover the benefits of physical therapy for sustainable development.

For example, a behavioral change towards health-enhancing levels of daily physical activity could be key for health as well as for the environment. A shift from car travel to any other mode of transport has been found to reduce the risk of non-communicable diseases^7^ and at the same time contribute to improved air quality, which will in turn reduce the burden of disease and number of deaths from air pollution.^8^^,^^9^ In fact, the WHO Global Action Plan for Physical Activity 2018–2030 emphasizes that measures taken to promote physical activity can contribute to achieving no less than 13 of the 17 SDGs.^10^ Put in other words, behaviors that favor health are often accompanied by benefits for the environment (Fig. 1, blue arrows). Focusing on the links between environment and health could be an important driving force towards a more sustainable development. For instance, climate policies have been found to generate a wide range of societal co-benefits with positive health effects,^11^ and the Lancet Commission on Health and Climate Change has estimated that measures taken to tackle climate change may provide the greatest global health opportunity of this century.^2^

At the same time, the health sector requires considerable energy and other resources to deliver its services and accounts for a substantial proportion of total greenhouse gas emissions worldwide^12^^,^^13^ (Fig. 1, black arrow). Emissions of greenhouse gases are mainly related to medical retail, hospitals, and ambulatory care.^12^ Also, pharmaceuticals and their active substances have become a global and common pollutant in aquatic systems, affecting all parts of the ecosystem.^14^

**Figure 2:**
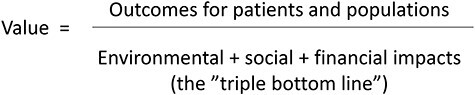
Sustainable value in health care. Reproduced with permission from Mortimer et al, 2018.^15^

Physical therapists, with expertise in promoting physical activity and empowering people for self-care and healthy lifestyles using non-pharmacological treatment methods, have the opportunity to be at the forefront of these issues. Since physical therapy research of today mainly focuses on evaluations of treatment outcomes, sometimes also involving measures of health economics, the value of physical therapy interventions could be strengthened, and interventions further refined, if outcomes of sustainable development are included. A model presented by the Centre for Sustainable Healthcare proposes such a perspective when measuring the value of health care.^15^^,^^16^ According to the model, the value of care is more than just treatment outcomes in relation to financial costs; all 3 dimensions of sustainable development should be recognized. This more holistic definition of value is illustrated as an equation (Fig. 2), where the outcomes for patients and populations are considered against a “triple bottom line” of environmental, social, and financial impacts or costs.^16^

A measure widely used for evaluating environmental impact (Fig. 2) is the sum of greenhouse gas emissions due to a given process, commonly referred to as a carbon footprint.^17^ A carbon footprint can be estimated by converting data on activities, such as travel and consultations, into kilograms of carbon dioxide equivalents and has been recognized as a standard for evaluating the environmental impact of health care.^18^ To date, there are only a few examples of original studies comparing the carbon footprint between different health care interventions such as comparing telemedicine consultations with clinical visits^19^ and providing dialysis in patient homes vs at the clinic.^20^ These examples of the operationalization of carbon footprinting in clinical research are relevant when identifying ways to evaluate the environmental impact of physical therapy interventions. However, examples from physical therapy research are, to our knowledge, still lacking. Although being recognized as the most urgent threat to human health, climate change is not the only environmental consideration, and other outcomes of environmental impact, such as pharmacological consumption, could be highly relevant for evaluation in physical therapy research.

The social impact in the triple bottom line (Fig. 2) concerns the social circumstances of patients, carers, staff and communities, also taking into account those affected by activities in the supply chain,^16^ but there is no standardized way of evaluation in the context of health care. When considering measures for evaluation, dimensions related to social determinants of health^21^ could be of relevance, and outcomes such as work and working conditions, sick leave, stress, social support, empowerment, participation, and health-related quality of life have been suggested as indicators.^22^ Recently, some of these outcomes have been used for evaluation of social impact in a health care context applying the triple bottom line.^23^

Regarding the financial costs in the triple bottom line (Fig. 2), evaluations of cost-effectiveness of health care interventions, including physical therapy, have developed over several decades and health economics is regarded an academic discipline in its own right, facilitating informed decision-making for clinicians and policymakers regarding clinical care and resource allocation.^24^

Physical therapy and its various methods are central to the health of individuals, but with this wider perspective we see a number of other, societal as well as environmental, co-benefits ready to be evaluated and communicated.

Global partnerships are described as essential to reach the goals for sustainable development,^5^ and for this paradigm shift to be implemented we believe that partnerships and policies need to be considered within physical therapy organizations and institutions at different levels, ie, globally, nationally, and locally as well as in collaboration with a range of various stakeholders. Further, funding agencies and scientific journals could create incentives by requesting a perspective of sustainable development to be included in research projects for obtaining funding or for publishing research papers.

However, to include a perspective of sustainable development in physical therapy research inevitably places new knowledge demands on physical therapists. This will be an important issue to address in physical therapy education at all levels, and it requires national, international, as well as interdisciplinary collaborations. The national and global physical therapy associations constitute excellent platforms for such knowledge sharing.

In a declaration from 2017 on health and climate change, the World Medical Association reached out for multiprofessional collaborations for a more sustainable health care.^25^ Thus, other health professions have commenced mobilization, and physical therapy could benefit from, as well as contribute to, such collaborations for change and impact, given that physical therapy has the potential to be a cornerstone in a more sustainable health care.

By this call, we hope to empower physical therapists to commence the work to identify and evaluate measures of sustainability relevant to their specific field of research, thereby including a perspective of sustainable development in research. Further, we hope to empower physical therapists worldwide to consider the opportunity for our profession to contribute in the transition towards a more sustainable health sector.
